# Origins of Biodiversity

**DOI:** 10.1371/journal.pbio.2000724

**Published:** 2016-11-02

**Authors:** Michael J. Benton

**Affiliations:** School of Earth Sciences, University of Bristol, Bristol, United Kingdom

## Abstract

Biodiversity today is huge, and it has a long history. Identifying rules for the heterogeneity of modern biodiversity—the high to low species richness of different clades—has been hard. There are measurable biodiversity differences between land and sea and between the tropics and temperate-polar regions. Some analyses suggest that the net age of a clade can determine its extinction risk, but this is equivocal. New work shows that, through geological time, clades pass through different diversification regimes, and those regimes constrain the balance of tree size and the nature of branching events.

Biodiversity is commonly understood as the number of species on Earth, sometimes more exactly termed “global species richness.” To understand modern biodiversity, it would be interesting to determine whether there are any “rules” or, at least, broad principles. Why, for example, are some groups, such as beetles or birds, so rich in species, whereas others, such as apes and ginkgos, are not? Are there any characteristics that increase or decrease the risk of extinction? What is the balance of innate characteristics, such as innovations, and external drivers, such as climate change, in determining the fates of species and larger groups?

## History of Biodiversity

It has been startlingly difficult to envisage the shape of increasing biodiversity through time for all sorts of reasons. The data come both from phylogenies based on living organisms and from the fossil record. However, even setting the level of modern global species richness is tricky. Estimates have ranged from 2–100 million species on Earth today, with current estimates at the lower end of this range, perhaps from 2–8 million [1]. These estimates depend on the fact that only 1.8 million distinct species of modern animals, plants, fungi, and microbes have been named, and various means of extrapolation then provide the higher estimates for the true number. Nonetheless, this sets the two margins of the plot of diversification through time as zero at the left-hand margin (some time in the dim, distant past) and, say, 5 million on the right-hand (present day) margin.

Such a plot depends on a key assumption that all life is related, and the sum of biodiversity through time can be documented as a single curve. Charles Darwin [2] identified the first principle of the origin of modern biodiversity, namely that all species were linked in a single great phylogeny, or tree of life, and that all could be traced back to a presumed single original species at some distant time in the geological past. The origin of life is now dated at 3.5–4 billion years ago, deep in the Precambrian.

**Fig 1:**
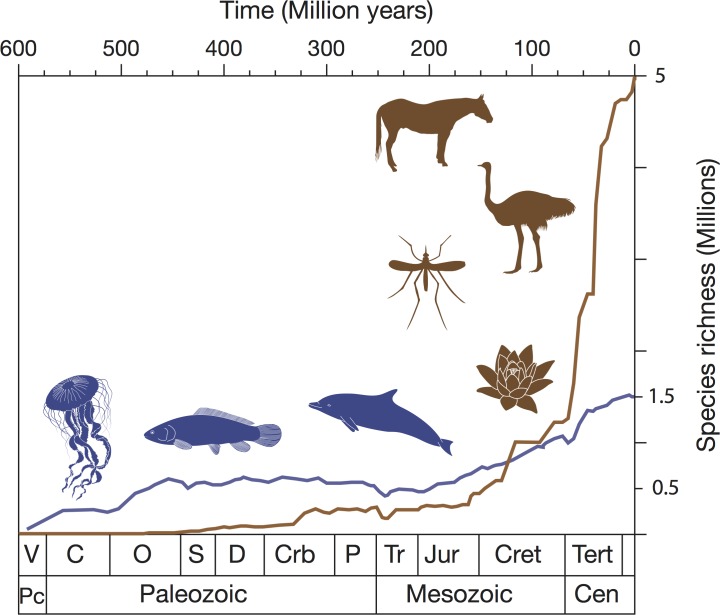
The history of biodiversity on land and in the sea. Note the postulated cross-over 125 million years ago, when life on land (brown line) became more diverse than life in the sea (blue line). The species-level plots are extrapolated from family-level plots in [5], and ideas expressed in [10]. Abbreviations: C, Cambrian; Crb, Carboniferous; Cret, Cretaceous; D, Devonian; J, Jurassic; O, Ordovician; P, permian; S, Silurian; Tert, Tertiary; Tr, Triassic; V, Vendian. Drafted by Simon Powell (University of Bristol).

The curve from a single species in the past to many millions today could have followed a variety of trajectories, ranging from a straight line to an exponential or even a logistic curve, the last implying that modern species richness levels were achieved at some point in the geological past and were maintained at a dynamic equilibrium level since. The narrative evidence from palaeontology points to an irregular global species diversification trajectory from one to many, with many setbacks from mass extinctions and other crises along the way. The underlying curve of increase does not show a long-term equilibrium and, if anything, looks exponential, both for life in the sea and life on land (Fig 1). This empirical pattern of increase [3–6] has proved robust to repeated analysis and addition of data.

There has been a long-running controversy among palaeontologists about whether such a curve can be read at face value, or whether there are major geological and sampling biases that mean we massively underestimate global biodiversity backwards in time [7,8]. However, the data screening and numerical manipulations required to achieve a pattern of long-term equilibrium since the Ordovician, some 500 million years ago (Mya), are so substantial as to cast doubt on those methods [9–11]. Arguments in favour of major biasing effects have been in terms of the “pull of the Recent” [7], all the factors that enable us to identify more and more species towards the present day, and so-called “megabias,” the suggestion that the empirical curve is entirely misleading [12]. However, it turns out that the pull of the Recent is a modest effect, representing about 5% of the Neogene data for bivalves [13], as well as for other groups, and many of the recent claims of megabias in sectors of the tetrapod fossil record are based on some false assumptions [14]. Therefore, palaeontologists all accept that the fossil record is full of holes, but they can still choose to accept the empirical curve as more or less correct or to prefer some kind of modified curve, but there is no consensus on what that modified curve might be.

Here, we accept the empirical curve as probably closer to the truth than the long-term equilibrium curve. Then, it can be highly informative to distinguish between life on land and life in the sea. A key point emerges from this comparison (Fig 1), which is that life in the sea was the major component initially but was overtaken by life on land at some point. The narrative of the history of life shows that algae, plants, and animals moved onto land at various points in the Palaeozoic, from 500–350 Mya, and during the age of the dinosaurs, life in the sea may still have held the upper hand. However, today, it is estimated that somewhere between 85% and 95% of species are terrestrial. This results from the huge dominance of insects today, especially beetles and social insects, as well as some other species-rich clades such as angiosperms, the flowering plants, and some tetrapod groups such as birds, lizards, and rodents. Various lines of evidence suggest that the cross-over happened 100–125 Mya, in the Early Cretaceous [10,15], perhaps connected with the Cretaceous Terrestrial Revolution [16], when the rise of angiosperms stimulated an explosion in diversity among insects and insect-eating birds, mammals, and lizards.

## Rules for Biodiversity

One of the first “rules of biodiversity” to be investigated concerns lineage age and extinction risk: are “ancient” lineages more or less likely to survive than newly emerged lineages? If this can be resolved, then there would be a means to estimate one factor in the extinction risk of living taxa. In his seminal paper on the Red Queen hypothesis, Van Valen [17] suggested that, for any major group, there was an equal chance of extinction for both long-lived and short-lived species and genera, his “Law of Constant Extinction.” This “law” has been disputed [18,19], however, and evidence presented for an age-dependent effect in which extinction risk is inversely related to genus age in most cases. This is still debated, and it could be argued logically that newly emerging species would be at highest risk as they are not adapted to resist competition, or, indeed, that long-established species might be more at risk from changing environments through having arisen in different times and different conditions [20]. Problems in resolving this seemingly simple question relate to the quality of fossil-based databases, definitions of genera and species, and the possibility of different behaviour of species during times of crisis and recovery from mass extinctions; nonetheless, there does seem to be evidence for some age dependency of species extinction risk, even though it ranges from positive to negative with respect to clade age.

**Fig 2:**
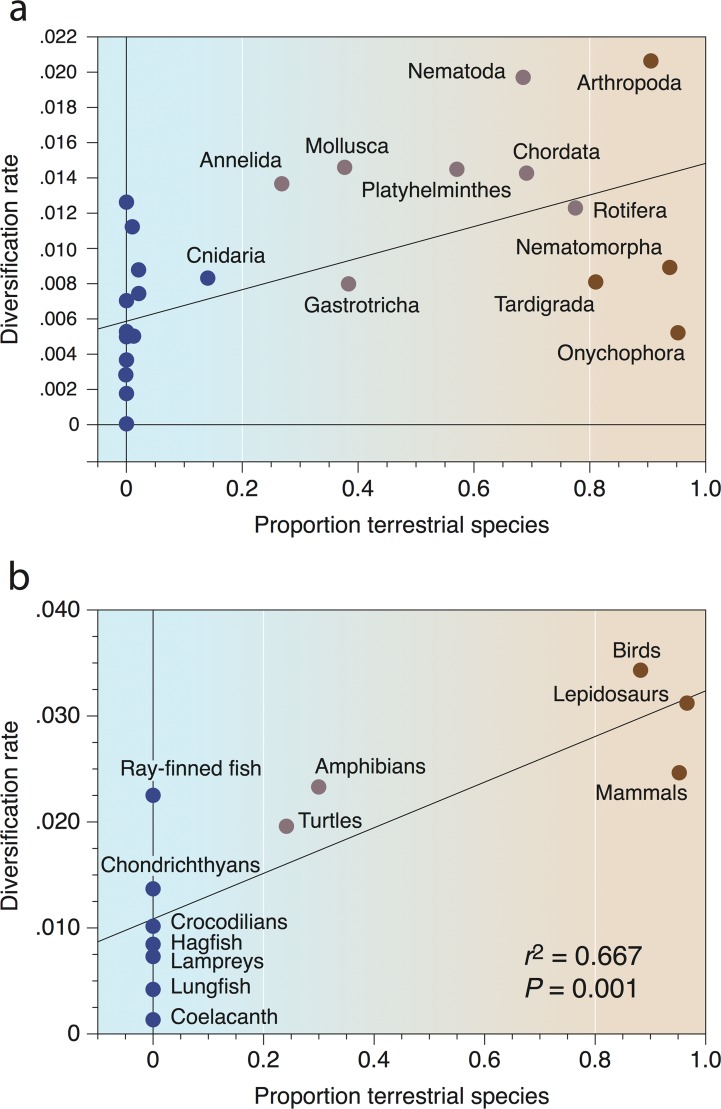
Relationship between habitat (proportion of terrestrial species) and net diversification rates. Data are shown for animals (a) and vertebrates (b). Predominantly marine forms are highlighted blue, and predominantly terrestrial forms, brown. Based on data in [23] and [24], respectively; drafted by Simon Powell (University of Bristol).

A second biodiversity “rule” may be the land–sea difference in diversification—why have terrestrial lineages shown such seemingly rapid diversification in the past 100 million years (Myr) (Fig 1)? One set of hypotheses is framed in ecological terms: perhaps marine species are constrained by limits of oceanic productivity, lower habitat heterogeneity, and lower endemism because of fewer biogeographic barriers than on land [10,21,22]. In a series of phylogenetically based tests, John Wiens has demonstrated that predominantly terrestrial animal clades have more rapid rates of diversification than marine clades [23], and this is also true for vertebrates alone [24]. The causes for this difference (Fig 2) have yet to be tested, but habitat architecture may trump climate change or physiology.

The third, very well-known biodiversity rule relates to the latitudinal diversity gradient. It is widely understood that life around the equator is more species rich and shows higher diversification rates than temperate or polar life; somehow, the heat of the air and sea leads to species-rich rain forests and reefs. Among mammals, tropical clades show high speciation rates, low extinction rates, or both, and the tropics then act as both a repository and driving force of biodiversity overall [25]. However, such an effect is not found among New World land birds, in which variations in speciation rate and extinction rate are not linked to latitude [26]. More work is required to determine whether the latitudinal diversity gradient is true for all clades. In cases of mixed marine–terrestrial clades, it is probably less important than the land–sea rule.

## Diversification Modes through Time

The comparative studies just described assume a single model through the entire history of each clade. However, evolutionary models may change during the lifetime of a major group, from its first emergence and filling of ecospace through its more mature phases in which new species may specialize and fill gaps or changing environments may lead to extinction or longer-term shifts. Some even argue for long-term slowdown in diversification rates as clades age, but that is controversial [27,28].

In their new paper, Eric Lewitus and Hélène Morlon [29] take this idea forward with a novel non-parametric meta-analysis of 214 family-level phylogenetic trees of vertebrates. They identify five key modes of diversification in this sample of trees, reflecting trade-offs in tree size and the heterogeneity and stem-to-tip distribution of branching events. Furthermore, during the history of a clade, it traverses phylogenetic space, reflecting a succession of dominant diversification modes, generally starting with the diversity-dependent model and passing through two further models before reaching the mass extinction type.

Not all evolutionary models are possible: the sample of vertebrate trees occupies only one-third of the volume of possible tree space. This points to constraints that probably exclude the possibility for any phylogeny to occupy those empty zones. An important point that emerges is that “biodiversity might well follow, if not entirely predictable, at least constrained trajectories through evolutionary time” [29, page 6]. These authors discriminate “tippy” and “stemmy” trees—those that show most branching near the tips (later) or near the stem (earlier) in clade history. Most vertebrate clades tend towards one of three main types: tippy trees; stemmy, irregular trees; and large, regular trees—and these are seen as “fitness optima.” Clades that diversify only early in their history (too stemmy) go extinct. Likewise, trees that expand and become too irregular (accelerated diversification in one subclade or multiple slowdowns in diversification) also experience contracted clades or extinction.

The study [29] highlights age-related differences between clades. Young clades tend to show early burst patterns followed by declining speciation rates, whereas older clades show more complex patterns as a result of subsequent opportunities for second or third expansion phases following mass extinctions or other opportunities. There are also differences among major clades, with mammals standing at odds to amphibians, reptiles, and birds. Mammals show few examples of tippy trees, and most are classed as type IV, reflecting temperature-dependence of speciation rate: a mode rarely seen among the other vertebrates.

Biodiversity is a core concept in biology and has been the subject of discussion for decades. It is remarkable that so few generalities about biodiversity exist—probably, as ever in biology, laws are elusive because of the contingent nature of the properties and history of each species and larger group. New phylogenetic comparative methods are now allowing some powerful analytical and meta-analytical studies of large-scale processes of macroevolution. Laws may not exist in biology, but there are generalities or rules, and these can be informative for determining our place on Earth, the future of biodiversity, and conservation policy planning.

## Abbreviations

Mya: million years ago
Myr: million years
